# Blood urea nitrogen to albumin ratio and mortality in critically ill patients with cardiogenic shock

**DOI:** 10.1371/journal.pone.0352659

**Published:** 2026-07-17

**Authors:** Xu Zhu, Qiong Wu, Zuo Kang, Shu Chen, Zhengdong Hu

**Affiliations:** 1 School of Integrated Chinese and Western Medicine, Hunan University of Chinese Medicine, Changsha, Hunan, China; 2 School of Humanities and Management, Hunan University of Chinese Medicine, Changsha, Hunan, China; International University of Health and Welfare, School of Medicine, JAPAN

## Abstract

**Purpose:**

Given the high mortality associated with cardiogenic shock (CS), this study aimed to evaluate the association between the blood urea nitrogen-to-albumin ratio (BAR) and mortality in critically ill patients with CS.

**Patients and methods:**

The data of this retrospective study were extracted from the Medical Information Mart for Intensive CARE-IV (MIMIC-IV v2.2) database. Patients who were diagnosed with CS were included as the study population. The primary outcome was one-year mortality, and secondary outcomes included in-hospital mortality and ICU mortality. Cox regression models were used to estimate the association between BAR and mortality.The prognostic value of BAR was assessed using the time-dependent area under the curve (AUC), net reclassification improvement (NRI), and integrated discrimination improvement (IDI).

**Results:**

A total of 1,177 patients were enrolled in the study. After adjustment for covariates, BAR was associated with one-year mortality (HR: 1.02 [1.01–1.03], *P* < 0.001 per 1-unit increase). Compared with the low-BAR group, the medium- and high-BAR groups showed higher risks of one-year mortality (medium group: HR 1.30 [1.02–1.66], *P* = 0.034; high group: HR 1.75 [1.32–2.33], *P* < 0.001). Similar associations were observed for in-hospital mortality (per 1-unit increase: HR 1.02 [1.01–1.04], *P* < 0.001; high vs. low group: HR 1.51 [1.08–2.13], *P* = 0.017) and ICU mortality (per 1-unit increase: HR 1.02 [1.01–1.04], *P* < 0.001; high vs. low group: HR 1.67 [1.17–2.40], *P* = 0.005). E‑value analysis suggested robustness to unmeasured confounding. BAR demonstrated favorable discriminative ability, with a time‑dependent AUC of 0.612 and a Harrell’s C‑index of 0.607. When combined with existing prognostic indicators, BAR significantly improved prognostic accuracy.

**Conclusion:**

In this study, higher BAR levels were associated with increased mortality in CS patients. BAR provided incremental prognostic value when combined with existing indicators.

## 1. Introduction

Cardiogenic shock (CS) is characterized by critically low cardiac output, leadings to severe end-organ hypoperfusion and hypoxia. This acute cardiovascular emergency often presents as multi-organ dysfunction syndrome (MODS), typically compounded by systemic inflammation with profound cellular and metabolic disturbances [1–3]. The primary etiological triad of CS comprises acute myocardial infarction, myocarditis, and acute decompensation of heart failure. Although early revascularization has significantly improved clinical outcomes, mortality rates remain substantial, ranging from 27% to 51% [2–5]. Given the poor prognosis associated with CS, identifying reliable prognostic indicators remains a critical clinical priority.

Albumin supports the body in multiple ways, from regulating fluid balance and fighting oxidative stress to easing inflammation. This makes it both a reliable indicator of nutritional status and a key player in maintaining overall organ health [6–7].. Similarly, blood urea nitrogen (BUN) serves as an important indicator, as its elevation is closely tied to critical states like renal hypoperfusion, low cardiac output, dehydration, and neurohormonal activation [8–9].In recent studies, the blood urea nitrogen-to-albumin ratio (BAR) has been reported as a predictor of mortality in intensive care unit (ICU) patients [10–13]. However, its prognostic value in patients with CS remains unevaluated. The high mortality rate of CS underscores the critical need for prognostic indicators. This study therefore aimed to explore the association between BAR and mortality in patients with CS.

## 2. Methods

### 2.1. Database

The data for this retrospective study were obtained from the Medical Information Mart for Intensive Care-IV (MIMIC-IV v2.2) database. The establishment of this database was approved by the Institutional Review Boards of the Massachusetts Institute of Technology and Beth Israel Deaconess Medical Center. Accordingly, the secondary analysis of its de-identified data in this study did not require separate ethical approval. The database includes information on patients admitted to ICUs at Beth Israel Deaconess Medical Center from 2008 to 2019. It contains comprehensive clinical data such as vital signs, laboratory measurements and medications [14–16]. One author (Xu Zhu) obtained access to the database and was responsible for the data extraction (certification number: 41711250).

### 2.2. Selection of participants

This study included all adult patients (≥18 years) diagnosed with CS during hospitalization, based on the International Classification of Diseases, Ninth or Tenth Revision (ICD-9/10) codes. Patients were excluded if they met any of the following criteria: (1) repeated ICU admission during the same hospitalization, (2) length of ICU stay less than 1 day, (3) missing data for BAR or mortality outcomes, or (4) missing data for all covariates included in the multivariable analysis.

### 2.3. Data extraction

Data extraction was performed using PostgreSQL (version 9.6). The variables extracted were demographic data (admission age, sex, ethnicity, admission weight), comorbidities (myocardial infarction, congestive heart failure, hypertension, diabetes, chronic kidney disease, sepsis), laboratory measurements (BUN, albumin, creatinine, lactate, eGFR, calcium, chloride, sodium, potassium, anion gap, bicarbonate, platelet, white blood cell, red blood cell, hemoglobin, glucose), disease severity scores (sequential organ failure assessment score, SOFA score), and treatments (fluid input, mechanical ventilation, renal replacement therapy, antibiotic drugs, vasopressors).

All laboratory measurements were defined as the first values recorded within 24 hours of ICU admission. The variable fluid input represented the total volume of infusion within the first 24 hours of ICU admission. Vasopressors included dobutamine, dopamine, epinephrine, norepinephrine, phenylephrine, vasopressin, and milrinone. We also collected hospital and ICU admission/discharge times as well as the date of death. For any variable measured multiple times, the first available value after ICU admission was used in the analysis..

The primary outcome was one-year mortality after ICU admission. Secondary outcomes were in-hospital mortality and ICU mortality. The time origin for all outcomes was defined as the time of ICU admission. For the one-year mortality analysis, patients were censored at one year after ICU admission if alive. For the ICU mortality analysis, patients were censored at ICU discharge if alive. For the in-hospital mortality analysis, censoring occurred at hospital discharge if alive. The Blood Urea Nitrogen to Albumin Ratio (BAR) was calculated as BUN (mg/dL) divided by albumin (g/dL).

### 2.4. Statistical analysis

Study participants were divided into three groups based on BAR tertiles. Continuous variables were described as mean ± standard deviation or (interquartile range, IQR) and compared using one-way ANOVA or the Kruskal-Wallis test, as appropriate. Categorical variables were presented as number (percentage) and compared using the chi-square test. Kaplan-Meier curves with the log-rank test were used to compare one-year mortality across BAR tertile groups.

Cox proportional hazards regression was used to quantify hazard ratios (HRs) and 95% confidence intervals (CIs) for the association between BAR and mortality. The proportional hazards assumption was tested using Schoenfeld residuals. Given that the main objective was to estimate the average association over the entire follow-up period, standard Cox models were used when the assumption was satisfied; when it was violated, Cox models with robust standard errors were employed to ensure valid inference [17].

We constructed two adjusted models: Model I adjusted for age, sex, and ethnicity; Model II additionally adjusted for weight, comorbidities, laboratory measurements, disease severity scores, and treatments. The complete list of variables adjusted for in Model II is provided in the Data Extraction section.

The discriminative and incremental prognostic value of BAR was evaluated using the time-dependent area under the receiver operating characteristic curve (AUC), integrated discrimination improvement (IDI), and net reclassification improvement (NRI).

Subgroup analyses were performed using stratified Cox regression by admission age, sex, comorbidities, and SOFA score. Interaction terms were used to explore heterogeneity across subgroups. The potential for unmeasured confounding was assessed by calculating the E-value [18]. A two-tailed P-value < 0.05 was considered statistically significant. All analyses were performed using R software (version 4.3.0) and Empower(R).

## 3. Results

### 3.1. Baseline characteristics

**Fig 1:**
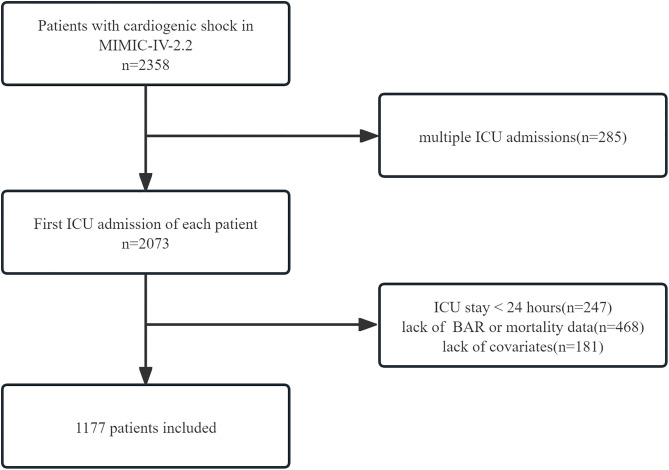
Flow chart.

A total of 1,177 patients were enrolled in the study (Fig 1). The mean age was 69.77 ± 14.63 years, 40.70% of the patients were female and 63.13% were White. Baseline characteristics stratified by BAR tertile are presented in Table 1. Significant differences across BAR tertiles were observed in age, ethnicity, congestive heart failure, hypertension, diabetes, chronic kidney disease, sepsis, creatinine, lactate, eGFR, chloride, sodium, potassium, anion gap, bicarbonate, platelets, red blood cell count, hemoglobin, glucose, SOFA score, fluid input, use of mechanical ventilation, and renal replacement therapy (all *P* < 0.05).

**Table 1:** Baseline characteristics of CS patients.

BAR group	All patients (0.69 - 58.75)	Low (0.69-7.40)	Medium (7.41,13.64)	High (13.67,58.75)	*P* value
Participants, No.	1177	392	390	395	
Age (years)	69.77 ± 14.63	65.82 ± 15.93	70.24 ± 14.12	73.22 ± 12.73	<0.001
Sex [female, n (%)]	479 (40.70%)	175 (44.64%)	155 (39.74%)	149 (37.72%)	0.127
Ethnicity[n (%)]					0.011
White	743 (63.13%)	223 (56.89%)	265 (67.95%)	255 (64.56%)	
Black	111 (9.43%)	37 (9.44%)	34 (8.72%)	40 (10.13%)	
Other	323 (27.44%)	132 (33.67%)	91 (23.33%)	100 (25.32%)	
Weight	82.62 ± 22.46	81.87 ± 22.33	83.88 ± 22.73	82.14 ± 22.33	0.396
Comorbidities [n (%)]					
Myocardial infarction	569 (48.34%)	208 (53.06%)	181 (46.41%)	180 (45.57%)	0.071
Congestive heart failure	919 (78.08%)	282 (71.94%)	305 (78.21%)	332 (84.05%)	<0.001
Hypertension	308 (26.17%)	126 (32.14%)	112 (28.72%)	70 (17.72%)	<0.001
Diabetes	444 (37.72%)	101 (25.77%)	147 (37.69%)	196 (49.62%)	<0.001
Chronic kidney disease	487 (41.38%)	85 (21.68%)	156 (40.00%)	246 (62.28%)	<0.001
Sepsis	355 (30.16%)	75 (19.13%)	129 (33.08%)	151 (38.23%)	<0.001
Laboratory measurements					
Creatinine (mg/dL)	1.50 (1.10-2.30)	1.00 (0.80-1.30)	1.50 (1.20-1.90)	2.50 (1.90-3.60)	<0.001
Lactate (mmol/L)	2.40 (1.50-4.00)	2.15 (1.40-3.80)	2.30 (1.50-4.00)	2.50 (1.70-4.15)	0.012
eGFR (mL/(min*1.73m^2^))	42.02 (24.11-63.82)	67.83 (49.94-86.44)	43.22 (31.56-56.20)	21.93 (14.05-30.87)	<0.001
Calcium (mg/dl)	8.41 ± 1.04	8.33 ± 1.06	8.50 ± 0.98	8.40 ± 1.09	0.075
Chloride (mmol/L)	100.97 ± 7.48	103.14 ± 7.05	100.83 ± 6.79	98.96 ± 7.96	<0.001
Sodium (mmol/L)	136.98 ± 5.97	137.93 ± 5.78	136.99 ± 5.24	136.02 ± 6.66	<0.001
Potassium (mmol/L)	4.62 ± 1.02	4.26 ± 0.82	4.63 ± 0.99	4.96 ± 1.11	<0.001
Anion gap (mmol/L)	18.51 ± 5.68	16.85 ± 5.02	18.30 ± 5.56	20.36 ± 5.89	<0.001
Bicarbonate (mg/dL)	20.55 ± 5.41	20.69 ± 4.36	21.09 ± 5.51	19.89 ± 6.15	0.006
Platelets (10^9^/L)	205.00 (146.00-272.00)	214.00 (158.75-278.00)	201.50 (148.00-268.00)	190.00 (132.50-264.00)	0.005
White blood cell (10^9^/L)	12.50 (8.70-17.40)	13.00 (8.78-18.35)	12.25 (8.72-17.40)	12.10 (8.70-16.00)	0.115
Red blood cell (10^12^/L)	3.82 ± 0.88	4.06 ± 0.88	3.83 ± 0.84	3.56 ± 0.83	<0.001
Hemoglobin (g/dL)	11.26 ± 2.60	12.10 ± 2.57	11.37 ± 2.51	10.32 ± 2.40	<0.001
Glucose (mg/dL)	154.00 (116.00-219.00)	159.50 (123.00-234.25)	152.00 (116.00-202.50)	147.00 (110.00-218.50)	0.033
Disease severity scores					
SOFA score	3.00 (1.00-5.00)	2.00 (0.00-4.25)	3.00 (1.00-5.00)	4.00 (1.00-6.00)	<0.001
Treatments [n (%)]					
Fluid input(L)	3.62(1.95-7.14)	4.02 (2.17-8.04)	3.91 (1.96-7.27)	3.07 (1.67-6.22)	<0.001
Mechanical ventilation[n (%)]	600 (50.98%)	231 (58.93%)	204 (52.31%)	165 (41.77%)	<0.001
Renal replacement therapy[n (%)]	283 (24.04%)	54 (13.78%)	98 (25.13%)	131 (33.16%)	<0.001
Antibiotic drugs[n (%)]	1017 (86.41%)	328 (83.67%)	348 (89.23%)	341 (86.33%)	0.076
Vasopressors[n (%)]	1067 (90.65%)	347 (88.52%)	356 (91.28%)	364 (92.15%)	0.189

All laboratory data were the first measurement values taken within 24 hours of admission to the ICU.

### 3.2. The association between BAR and outcomes

We employed Cox proportional-hazards regression models to assess the associations between BAR and one-year, in-hospital, and ICU mortality. The proportional hazards assumption was met in the in-hospital and ICU mortality models (*P* > 0.05 in fully adjusted models) but was violated in the one-year mortality model (*P* < 0.05). Therefore, hazard ratios for in-hospital and ICU mortality were obtained from standard Cox models, whereas estimates for one-year mortality were derived using a Cox model with robust standard errors. Results from the time-dependent Cox regression model are included as a sensitivity analysis (Table S1 in S1 File).

After full adjustment, each 1-unit increase in BAR remained associated with one-year mortality (HR: 1.02 [1.01–1.03], *P* < 0.001), in-hospital mortality (HR: 1.02 [1.01–1.04], *P* < 0.001), and ICU mortality (HR: 1.02 [1.01–1.04], *P* < 0.001). When analyzed by BAR tertiles and compared with the low-BAR group, higher BAR levels were associated with increased risk of one-year mortality (medium tertile: 1.30 [1.02–1.66], *P* = 0.034; high tertile: 1.75 [1.32–2.33], *P* < 0.001), in-hospital mortality (medium tertile: 1.16 [0.87–1.56], *P* = 0.311; high tertile: 1.51 [1.08–2.13], *P* = 0.017), and ICU mortality (medium tertile: 1.30 [0.96–1.77], *P* = 0.087; high tertile: 1.67 [1.17–2.40], *P* = 0.005) (Table 2).

**Table 2:** Univariate and multivariate results by cox regression.

Exposure	Non-adjusted HR (95%CI) *P*	Adjust I HR (95%CI) *P*	Adjust II HR (95%CI) *P*
One-year mortality			
BAR per-1unit	1.04 (1.03, 1.04) <0.001	1.03 (1.03, 1.04) <0.001	1.02 (1.01, 1.03) <0.001
BAR group			
Low	1.0	1.0	1.0
Medium	1.64 (1.33, 2.02) <0.001	1.59 (1.29, 1.97) <0.001	1.30 (1.02, 1.66) 0.034
High	2.62 (2.15, 3.19) <0.001	2.41 (1.97, 2.94) <0.001	1.75 (1.32, 2.33) <0.001
*P* for trend	1.62 (1.47, 1.78) <0.001	1.55 (1.40, 1.71) <0.001	1.33 (1.15, 1.53) <0.001
In-hospital mortality			
BAR per-1unit	1.03 (1.02, 1.04) <0.001	1.02 (1.01, 1.03) <0.001	1.02 (1.01, 1.04) <0.001
BAR group			
Low	1.0	1.0	1.0
Medium	1.28 (0.99, 1.66) 0.059	1.31 (1.01, 1.70) 0.042	1.16 (0.87, 1.56) 0.311
High	1.87 (1.46, 2.38) <0.001	1.78 (1.39, 2.28) <0.001	1.51 (1.08, 2.13) 0.017
*P* for trend	1.37 (1.22, 1.55) <0.001	1.34 (1.18, 1.51) <0.001	1.24 (1.04, 1.47) 0.014
ICU mortality			
BAR per-1unit	1.03 (1.02, 1.04) <0.001	1.02 (1.01, 1.03) <0.001	1.02 (1.01, 1.04) <0.001
BAR group			
Low	1.0	1.0	1.0
Medium	1.31 (1.00, 1.72) 0.046	1.36 (1.04, 1.79) 0.026	1.30 (0.96, 1.77) 0.087
High	1.88 (1.46, 2.43) <0.001	1.80 (1.38, 2.33) <0.001	1.67 (1.17, 2.40) 0.005
*P* for trend	1.38 (1.21, 1.57) <0.001	1.34 (1.18, 1.52) <0.001	1.29 (1.08, 1.55) 0.005

Non-adjusted model adjusted for none.

Adjust I model adjusted for ethnicity, admissionage, sex.

Adjust II model adjusted for model I plus weight, myocardial infarct, congestive heart failure, hypertension, diabetes, chronic kidney disease, sepsis, creatinine, lactate, eGFR, calcium, chloride, sodium, potassium, anion gap, bicarbonate, platelets, white blood cell, red blood cell, hemoglobin, glucose,SOFA score, fluid input, mechanical ventilation, renal replacement therapy, antibiotic drugs, vasopressors.

**Fig 2:**
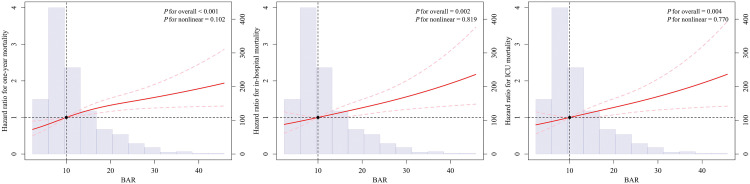
Dose-response relationships between BAR and mortality endpoints.

**Fig 3:**
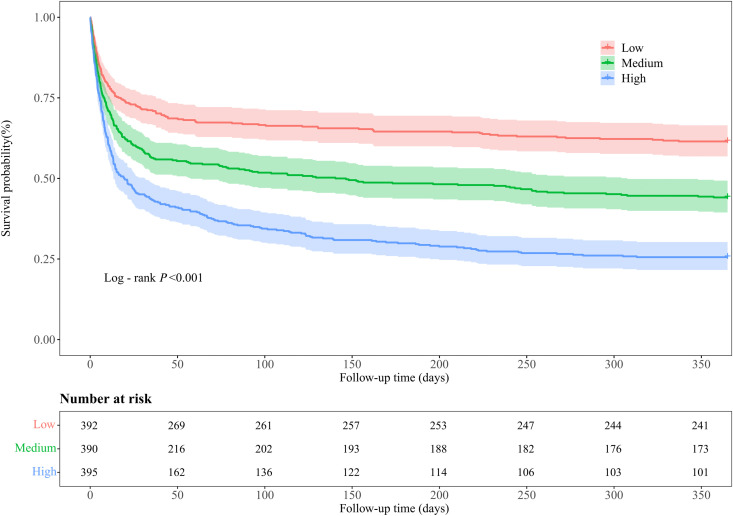
Kaplan-Meier curves for one-year mortality of BAR groups.

Fig 2 illustrates the dose-response associations between BAR and the three mortality outcomes. A positive dose-response relationship was observed for all endpoints. Kaplan-Meier curves for one-year mortality indicated that the high-BAR group had the lowest survival probability (log-rank *P* < 0.05) (Fig 3).

### 3.3. Discriminative and Calibrative Performance of BAR

**Fig 4:**
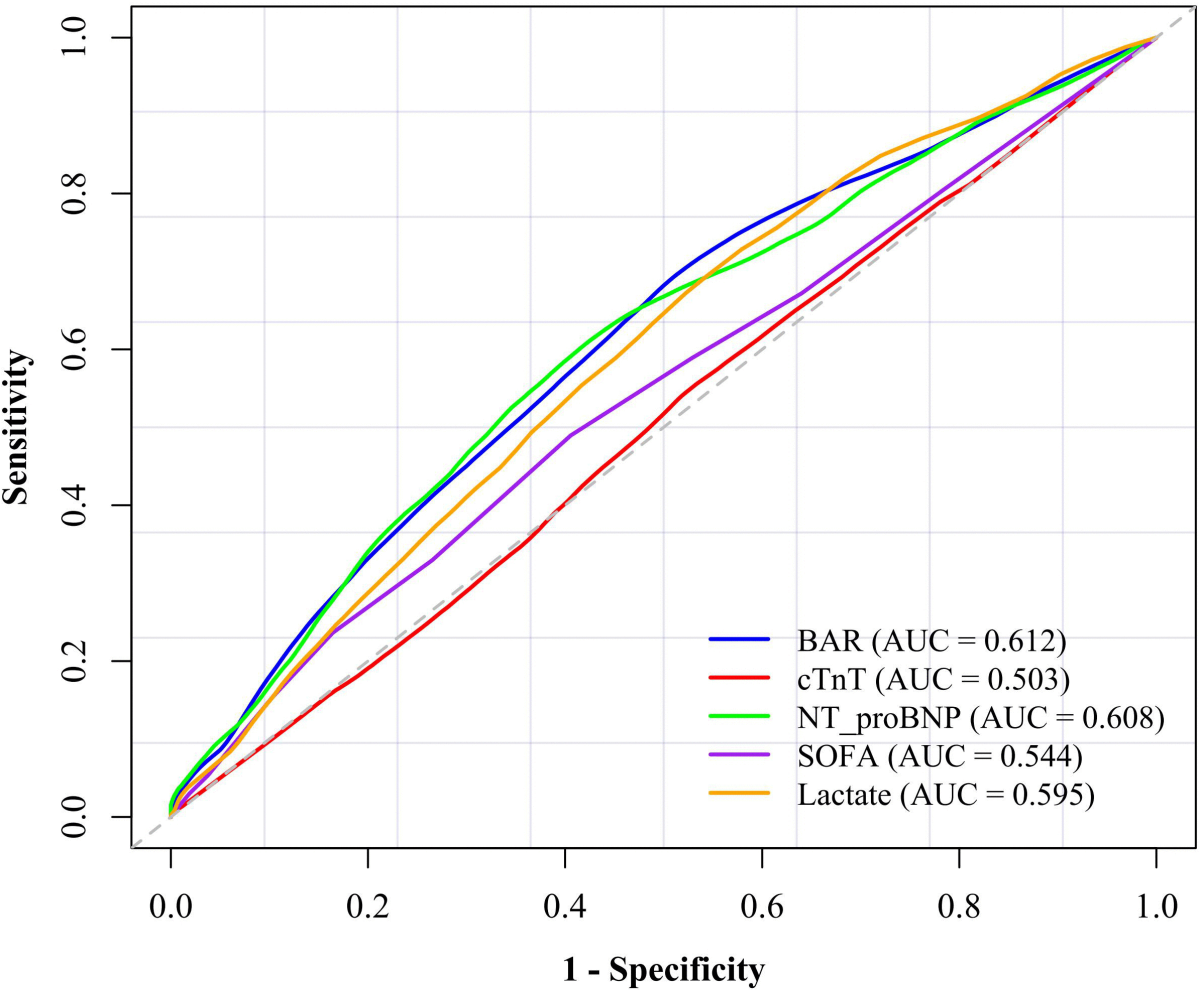
Time-dependent ROC curves for one-year mortality.

**Fig 5:**
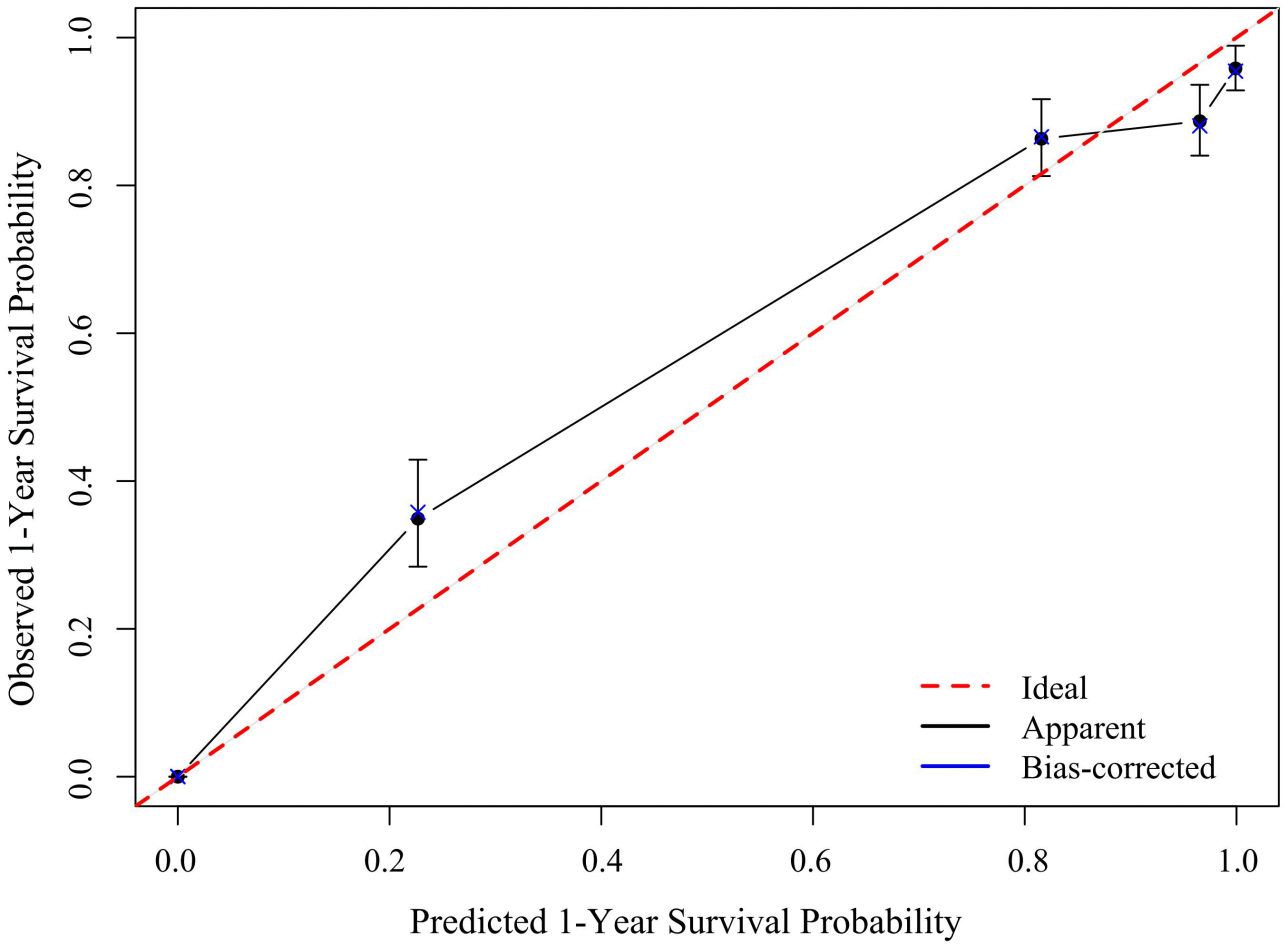
Calibration plot of the multivariable Cox model for one-year mortality.

To evaluate the discriminative ability of BAR, we calculated the time-dependent AUC for one-year mortality. BAR demonstrated an AUC of 0.612, which was comparable to NT-proBNP (0.608) and lactate (0.595), and higher than the SOFA score (0.544) and troponin T (0.503) (Fig 4). A consistent pattern was observed for Harrell’s C-index, with BAR (0.607) and lactate (0.605) showing similar concordance, followed by NT-proBNP (0.578), the SOFA score (0.543), and troponin T (0.500). The calibration plot of the multivariable Cox model indicated good agreement between observed and predicted probabilities (Fig 5). Time-dependent ROC curves for BUN, albumin, and BAR are presented in Figure S1 in S1 File.

### 3.4. Incremental clinical value of BAR

To evaluate the incremental prognostic value of BAR for one-year mortality over existing indicators, we calculated the net reclassification improvement (NRI) and integrated discrimination improvement (IDI). As shown in Table 3, BAR significantly improved prognostic performance when added to the SOFA score, lactate, troponin T, or NT-proBNP, compared to using these indicators alone.

**Table 3:** Incremental clinical value of BAR for one-year mortality.

	NRI	*P* for NRI	IDI	*P* for IDI
SOFA vs BAR+SOFA	0.481	<0.001	0.075	<0.001
Lactate vs BAR+Lactate	0.478	<0.001	0.080	<0.001
CTnT vs BAR + CTnT	0.475	<0.001	0.076	<0.001
NT-proBNP vs BAR + NT-proBNP	0.444	<0.001	0.046	<0.001

Specifically, adding BAR to the SOFA score resulted in an NRI of 0.481 (*P* < 0.001) and an IDI of 0.075 (*P* < 0.001). Similarly, BAR provided statistically significant improvements when added to lactate (NRI = 0.478, *P* < 0.001; IDI = 0.080, *P* < 0.001), troponin T (NRI = 0.475, *P* < 0.001; IDI = 0.076, *P* < 0.001), and NT-proBNP (NRI = 0.444, *P* < 0.001; IDI = 0.046, *P* < 0.001).

### 3.5. Sensitivity analysis

Subgroup analyses were performed based on admission age, sex, SOFA score, myocardial infarction, congestive heart failure, hypertension, diabetes, and chronic kidney disease using Cox models with robust standard errors. The association between BAR and one-year mortality was not statistically significant in the subgroups of age ≤ 65 years, myocardial infarction, hypertension, and diabetes. No significant interaction was observed in any subgroup (*P* for interaction > 0.05), indicating that the association between BAR and mortality was generally consistent across these patient characteristics (Table 4).

**Table 4:** Subgroup analysis for one-year mortality.

	No. of events/No. of patients	HR (95%CI) *P*	*P* for interaction
Age			0.816
≤ 65	162/396	1.03 (1.00, 1.05) 0.059	
> 65	501/781	1.02 (1.01, 1.03) <0.001	
Sex			0.404
Female	280/479	1.02 (1.01, 1.04) 0.003	
Male	383/698	1.03 (1.01, 1.04) <0.001	
SOFA score			0.472
≤ 3	343/658	1.02 (1.01, 1.04) 0.003	
> 3	320/519	1.03 (1.01, 1.04) <0.001	
Myocardial infarct			0.349
No	340/608	1.03 (1.02, 1.05) <0.001	
Yes	323/569	1.01 (1.00, 1.03) 0.135	
Congestive heart failure			0.335
No	153/258	1.03 (1.00, 1.05) 0.020	
Yes	510/919	1.02 (1.01, 1.03) <0.001	
Hypertension			0.589
No	511/869	1.03 (1.02, 1.04) <0.001	
Yes	152/308	1.00 (0.97, 1.03) 0.967	
Diabetes			0.607
No	394/733	1.03 (1.02, 1.04) <0.001	
Yes	269/444	1.01 (1.00, 1.03) 0.191	
Chronic kidney disease			0.900
No	336/690	1.03 (1.01, 1.05) 0.002	
Yes	327/487	1.02 (1.01, 1.03) 0.002	

To assess the potential impact of unmeasured confounding, an E-value analysis was conducted. The observed association appeared robust, with an E-value of 2.87 for the point estimate. The E-value for the lower confidence interval bound was 1.95, suggesting that to fully explain away the observed association, an unmeasured confounder would need to be associated with both BAR and one-year mortality by relative risks of at least 1.95.

## 4. Discussion

The Blood Urea Nitrogen to Albumin Ratio (BAR) has been associated with prognosis across various diseases, suggesting its broad utility as a prognostic indicator. For instance, BAR has been reported as a useful prognostic indicator for lung cancer patients in intensive care units [12], the best parameter for predicting outcomes in ICU patients with non-CKD illness [13], a powerful predictor of in-hospital mortality in older emergency department patients [19], and a risk factor for mortality in patients with hospital-acquired pneumonia [20].

In this study, we evaluated the association between BAR and outcomes in critically ill patients with CS. We found that BAR, both as a categorical and continuous variable, was significantly associated with one-year, in-hospital, and ICU mortality in multivariate regression analysis. In time-dependent ROC analysis, BAR demonstrated favorable and comparable discriminative ability. Furthermore, NRI and IDI analyses showed that BAR enhanced the accuracy of prognostic assessment. These findings suggest that BAR may serve as a supplementary prognostic indicator when SOFA score calculation is not feasible. Alternatively, established indicators such as the SOFA score, lactate, NT-proBNP, and troponin T can be used alongside BAR to improve risk stratification.

Previous studies have suggested that BUN levels correlate with short-, intermediate-, and long-term prognosis in patients with cardiovascular diseases, including heart failure [21–25]. In CS, low cardiac output and systemic hypoperfusion can lead to reduced renal blood flow, decreased glomerular filtration rate, and impaired urea excretion, thereby increasing BUN levels [26–28]. Additionally, decreased effective circulating volume due to factors such as sweating or vomiting may reduce urine flow and enhance tubular urea reabsorption, further elevating BUN concentration [29–31].

Albumin plays a vital role in maintaining osmotic pressure, regulating vascular permeability and acid-base balance, and functions as an anti-inflammatory and antioxidant molecule. Studies have shown that hypoalbuminemia is a common indicator of poor outcome in CS [32–33]. This condition is frequently complicated by a systemic inflammatory response, which can induce severe cellular and metabolic dysregulation. This inflammatory state may lower albumin levels by impairing hepatic protein synthesis and increasing capillary leakage [34–37].

Taken together, the pathways described above suggest that the prognostic relevance of BAR in CS may be understood through its reflection of two central pathophysiological processes: renal hypoperfusion and systemic inflammation. A high BAR indicates both an elevated BUN concentration, which is associated with reduced renal perfusion, and a low albumin level, which is linked to heightened inflammation.

The clinical implications of these findings should be considered alongside the strengths and limitations of our study. This study has several strengths. First, to our knowledge, it is the first to specifically investigate the association between BAR and mortality risk in critically ill patients with CS. Second, since both BUN and albumin are routinely measured with rapid, low-cost tests, BAR represents a feasible and practical indicator for prognostic assessment in clinical practice. Third, the analysis included 1,177 patients from the high-quality MIMIC database; this substantial sample size enhances the reliability of our findings. Finally, BAR primarily reflects a patient’s physiological reserve and metabolic homeostasis, thereby offering a perspective that complements established tools such as the CardShock score or SCAI staging, which focus more on shock severity and clinical progression.

Several limitations should be acknowledged. First, BAR in this study was calculated using only the first measurement after ICU admission. Since dynamic changes in biomarkers often carry greater prognostic value than single values [38–40], this approach may underestimate the true association between BAR and mortality. Second, we were unable to account for medications administered prior to ICU admission. Third, due to a high proportion of missing values (exceeding 10%), some variables were excluded from the multivariable regression analysis. Fourth, as a single-center retrospective study, the findings may be subject to selection bias; prospective studies are needed to validate these results. Fifth, key clinical data were unavailable in the database, including detailed etiology, SCAI shock classification, mechanical circulatory support, nutritional support, and hemodynamic parameters, which therefore could not be adjusted for in our analysis.

Our findings support BAR as a clinically accessible prognostic indicator that provides additional prognostic information. However, given the observational design, it should not yet guide therapeutic decisions. These findings require validation in prospective, multicenter studies to define BAR’s precise role in clinical management pathways.

## 5. Conclusions

In this retrospective cohort study, elevated BAR at ICU admission was independently associated with increased mortality in patients with CS. When added to existing prognostic indicators, BAR provided significant incremental prognostic value. While these findings highlight BAR’s potential as a supplementary prognostic indicator, its clinical utility requires prospective validation.

## Supporting information

S1 File This file contains Table S1 (The results of the time-dependent Cox regression model) and Figure S1 (Time-dependent ROC curves of BUN, albumin and BAR). (DOC)

## Abbreviations

BUN: blood urea nitrogen
WBC: white blood cell
RBC: red blood cell
SOFA: sequential organ failure assessment
BAR: blood urea nitrogen-to-albumin ratio
CS: cardiogenic shock
ICU: intensive care unit
HR: hazard ratio
CI: confidence interval

## References

[1] Kim JH, Sunkara A, Varnado S. Management of cardiogenic shock in a cardiac intensive care unit. Methodist Debakey Cardiovasc J. 2020;16(1):36–42. doi: 10.14797/mdcj-16-1-36 32280416 PMC7137626

[2] Iborra-Egea O, Rueda F, García-García C, Borràs E, Sabidó E, Bayes-Genis A. Molecular signature of cardiogenic shock. Eur Heart J. 2020;41(39):3839–48. doi: 10.1093/eurheartj/ehz783 31722370

[3] van Diepen S, Katz JN, Albert NM, Henry TD, Jacobs AK, Kapur NK, et al. Contemporary management of cardiogenic shock: a scientific statement from the American Heart Association. Circulation. 2017;136(16):e232–68. doi: 10.1161/CIR.0000000000000525 28923988

[4] Becher PM, Schrage B, Sinning CR, Schmack B, Fluschnik N, Schwarzl M, et al. Venoarterial extracorporeal membrane oxygenation for cardiopulmonary support. Circulation. 2018;138(20):2298–300. doi: 10.1161/CIRCULATIONAHA.118.036691 30571518

[5] Kolte D, Khera S, Aronow WS, Mujib M, Palaniswamy C, Sule S, et al. Trends in incidence, management, and outcomes of cardiogenic shock complicating ST-elevation myocardial infarction in the United States. J Am Heart Assoc. 2014;3(1):e000590. doi: 10.1161/JAHA.113.000590 24419737 PMC3959706

[6] Yu Y, Liu Y, Ling X, Huang R, Wang S, Min J, et al. The neutrophil percentage-to-albumin ratio as a new predictor of all-cause mortality in patients with cardiogenic shock. Biomed Res Int. 2020;2020:7458451. doi: 10.1155/2020/7458451 33294452 PMC7714577

[7] Don BR, Kaysen G. Serum albumin: relationship to inflammation and nutrition. Semin Dial. 2004;17(6):432–7. doi: 10.1111/j.0894-0959.2004.17603.x 15660573

[8] Roche M, Rondeau P, Singh NR, Tarnus E, Bourdon E. The antioxidant properties of serum albumin. FEBS Lett. 2008;582(13):1783–7. doi: 10.1016/j.febslet.2008.04.057 18474236

[9] Arihan O, Wernly B, Lichtenauer M, Franz M, Kabisch B, Muessig J, et al. Blood Urea Nitrogen (BUN) is independently associated with mortality in critically ill patients admitted to ICU. PLoS One. 2018;13(1):e0191697. doi: 10.1371/journal.pone.0191697 29370259 PMC5784990

[10] Richter B, Sulzgruber P, Koller L. Blood urea nitrogen has additive value beyond estimated glomerular filtration rate for prediction of long-term mortality in patients with acute myocardial infarction. Eur J Intern Med. 2019;59:84–90. 30072202 10.1016/j.ejim.2018.07.019

[11] Ryu S, Oh SK, Cho SU, You Y, Park JS, Min JH, et al. Utility of the blood urea nitrogen to serum albumin ratio as a prognostic factor of mortality in aspiration pneumonia patients. Am J Emerg Med. 2021;43:175–9. doi: 10.1016/j.ajem.2020.02.045 32122715

[12] Peng X, Huang Y, Fu H. Prognostic value of blood urea nitrogen to serum albumin ratio in intensive care unit patients with lung cancer. Int J Gen Med. 2021;14:7349–59. 34737629 10.2147/IJGM.S337822 PMC8560134

[13] Gundpatil DB, Somani BL, Saha TK, Banerjee M. Serum urea:albumin ratio as a prognostic marker in critical patients with non-chronic kidney disease. Indian J Clin Biochem. 2014;29(1):97–100. doi: 10.1007/s12291-012-0274-z 24478558 PMC3903933

[14] Johnson A, Bulgarelli L, Pollard T, Horng S, Celi LA, Mark R. MIMIC-IV (version 1.0). PhysioNet. 2021. doi: 10.13026/s6n6-xd98

[15] Liu T, Zhao Q, Du B. Effects of high-flow oxygen therapy on patients with hypoxemia after extubation and predictors of reintubation: a retrospective study based on the MIMIC-IV database. BMC Pulm Med. 2021;21(1):160. doi: 10.1186/s12890-021-01526-2 33985472 PMC8118109

[16] Zhou S, Zeng Z, Wei H. Early combination of albumin with crystalloids administration might be beneficial for the survival of septic patients: a retrospective analysis from MIMIC-IV database. Ann Intensive Care. 2021;11(1):42. 33689042 10.1186/s13613-021-00830-8 PMC7947075

[17] Lin DY, Wei LJ. The robust inference for the cox proportional hazards model. J Am Stat Assoc. 1989;84(408):1074–8. doi: 10.1080/01621459.1989.10478874

[18] Haneuse S, VanderWeele TJ, Arterburn D. Using the E-value to assess the potential effect of unmeasured confounding in observational studies. JAMA. 2019;321(6):602–3. doi: 10.1001/jama.2018.21554 30676631

[19] Dundar ZD, Kucukceran K, Ayranci MK. Blood urea nitrogen to albumin ratio is a predictor of in-hospital mortality in older emergency department patients. Am J Emerg Med. 2021;46:349–54. doi: 10.1016/j.ajem.2020.10.008 33069540

[20] Feng D-Y, Zhou Y-Q, Zou X-L, Zhou M, Yang H-L, Chen X-X, et al. Elevated blood urea nitrogen-to-serum albumin ratio as a factor that negatively affects the mortality of patients with hospital-acquired pneumonia. Can J Infect Dis Med Microbiol. 2019;2019:1547405. doi: 10.1155/2019/1547405 31316681 PMC6604473

[21] Kajimoto K, Minami Y, Sato N, et al. Investigators of the Acute Decompensated Heart Failure Syndromes (ATTEND) registry. Serum sodium concentration, blood urea nitrogen, and outcomes in patients hospitalized for acute decompensated heart failure. Int J Cardiol. 2016;222:195–201. 27497094 10.1016/j.ijcard.2016.07.255

[22] Kajimoto K, Sato N, Takano T. eGFR and outcomes in patients with acute decompensated heart failure with or without elevated BUN. Clin J Am Soc Nephrol. 2016;3(11):405–12. 10.2215/CJN.08210815 PMC4791821 26769764

[23] Ren X, Qu W, Zhang L. Role of blood urea nitrogen in predicting the post-discharge prognosis in elderly patients with acute decompensated heart failure. Sci Rep. 2018;8(1):13507. doi: 10.1038/s41598-018-31925-1 30202087 PMC6131513

[24] Richter B, Sulzgruber P, Koller L, Steininger M, El-Hamid F, Rothgerber DJ, et al. Blood urea nitrogen has additive value beyond estimated glomerular filtration rate for prediction of long-term mortality in patients with acute myocardial infarction. Eur J Intern Med. 2019;59:84–90. doi: 10.1016/j.ejim.2018.07.019 30072202

[25] Horiuchi Y, Aoki J, Tanabe K. A high level of blood urea nitrogen is a significant predictor for in-hospital mortality in patients with acute myocardial infarction. Int Heart J. 2018;59(2):263–71. 29459576 10.1536/ihj.17-009

[26] Zhu Y, Sasmita BR, Hu X, Xue Y, Gan H, Xiang Z, et al. Blood urea nitrogen for short-term prognosis in patients with cardiogenic shock complicating acute myocardial infarction. Int J Clin Pract. 2022;2022:9396088. doi: 10.1155/2022/9396088 35685591 PMC9159167

[27] Liu E-Q, Zeng C-L. Blood urea nitrogen and in-hospital mortality in critically ill patients with cardiogenic shock: analysis of the MIMIC-III database. Biomed Res Int. 2021;2021:5948636. doi: 10.1155/2021/5948636 33604376 PMC7870297

[28] Sun D, Wei C, Li Z. Blood urea nitrogen to creatinine ratio is associated with in-hospital mortality among critically ill patients with cardiogenic shock. BMC Cardiovasc Disord. 2022;22(1):258. doi: 10.1186/s12872-022-02692-9 35676647 PMC9178813

[29] van Diepen S, Katz JN, Albert NM, American Heart Association Council on Clinical Cardiology; Council on Cardiovascular and Stroke Nursing; and Mission: Lifeline. et al. Contemporary management of cardiogenic shock: a scientific statement from the American Heart Association. Circulation. 2017;136(16):e232–68. 10.1161/CIR.0000000000000525 28923988

[30] Yoo M, Bediako EO, Akca O. Syndrome of inappropriate antidiuretic hormone (SIADH) secretion caused by squamous cell carcinoma of the nasopharynx: case report. Clin Exp Otorhinolaryngol. 2008;1(2):110–2. doi: 10.3342/ceo.2008.1.2.110 19434282 PMC2671796

[31] Schrier RW. Blood urea nitrogen and serum creatinine: not married in heart failure. Circ Heart Fail. 2008;1(1):2–5. doi: 10.1161/CIRCHEARTFAILURE.108.770834 19808263

[32] Jäntti T, Tarvasmäki T, Harjola V-P, Parissis J, Pulkki K, Javanainen T, et al. Hypoalbuminemia is a frequent marker of increased mortality in cardiogenic shock. PLoS One. 2019;14(5):e0217006. doi: 10.1371/journal.pone.0217006 31095609 PMC6522037

[33] Oduncu V, Erkol A, Karabay CY, Kurt M, Akgün T, Bulut M, et al. The prognostic value of serum albumin levels on admission in patients with acute ST-segment elevation myocardial infarction undergoing a primary percutaneous coronary intervention. Coron Artery Dis. 2013;24(2):88–94. doi: 10.1097/MCA.0b013e32835c46fd 23249632

[34] Levitt DG, Levitt MD. Human serum albumin homeostasis: a new look at the roles of synthesis, catabolism, renal and gastrointestinal excretion, and the clinical value of serum albumin measurements. Int J Gen Med. 2016;9:229–55. 27486341 10.2147/IJGM.S102819 PMC4956071

[35] Eckart A, Struja T, Kutz A, Baumgartner A, Baumgartner T, Zurfluh S, et al. Relationship of nutritional status, inflammation, and serum albumin levels during acute illness: a prospective study. Am J Med. 2020;133(6):713–722.e7. doi: 10.1016/j.amjmed.2019.10.031 31751531

[36] Jensen GL, Mirtallo J, Compher C, Dhaliwal R, Forbes A, Grijalba RF, et al. Adult starvation and disease-related malnutrition: a proposal for etiology-based diagnosis in the clinical practice setting from the International Consensus Guideline Committee. Clin Nutr. 2010;29(2):151–3. doi: 10.1016/j.clnu.2009.11.010 20071059

[37] Peng Y, Huang W, Shi Z, Chen Y, Ma J. Positive association between systemic immune-inflammatory index and mortality of cardiogenic shock. Clin Chim Acta. 2020;511:97–103. doi: 10.1016/j.cca.2020.09.022 33045194

[38] Pardo E, Jabaudon M, Godet T, Pereira B, Morand D, Futier E, et al. Dynamic assessment of prealbumin for nutrition support effectiveness in critically ill patients. Clin Nutr. 2024;43(6):1343–52. doi: 10.1016/j.clnu.2024.04.015 38677045

[39] Cheng L, Zhang F, Xue W, Yu P, Wang X, Wang H, et al. Association of dynamic change of triglyceride-glucose index during hospital stay with all-cause mortality in critically ill patients: a retrospective cohort study from MIMIC IV2.0. Cardiovasc Diabetol. 2023;22(1):142. 37330498 10.1186/s12933-023-01874-9 PMC10276426

[40] Li D, Liu S, Zhang J, Cheng W, Mao J, Cui N. Exploring dynamic change in arterial base excess with patient outcome and lactate clearance in the intensive care unit by hierarchical time-series clustering. Front Med (Lausanne). 2022;9:1020806. 36425098 10.3389/fmed.2022.1020806 PMC9679290

