# Supplementary material for: Blood urea nitrogen to albumin ratio and mortality in critically ill patients with cardiogenic shock
Source: PLoS One. 2026 Jul 17;21(7):e0352659. doi: 10.1371/journal.pone.0352659 (PMC13379123; doi:10.1371/journal.pone.0352659)
Supplement: S1 File — (DOC) [file pone.0352659.s001.doc]

**1.The results of the time-dependent Cox regression model**

Table S1 The results of the time-dependent Cox regression model

| Main_effect_coef.bar | Interaction_coef.tt(bar) | Interaction *P* |
| --- | --- | --- |
| 0.003514011 | 0.007866954 | 0.004216596 |

**2.Time-dependent ROC curves of BUN, albumin and BAR**

| 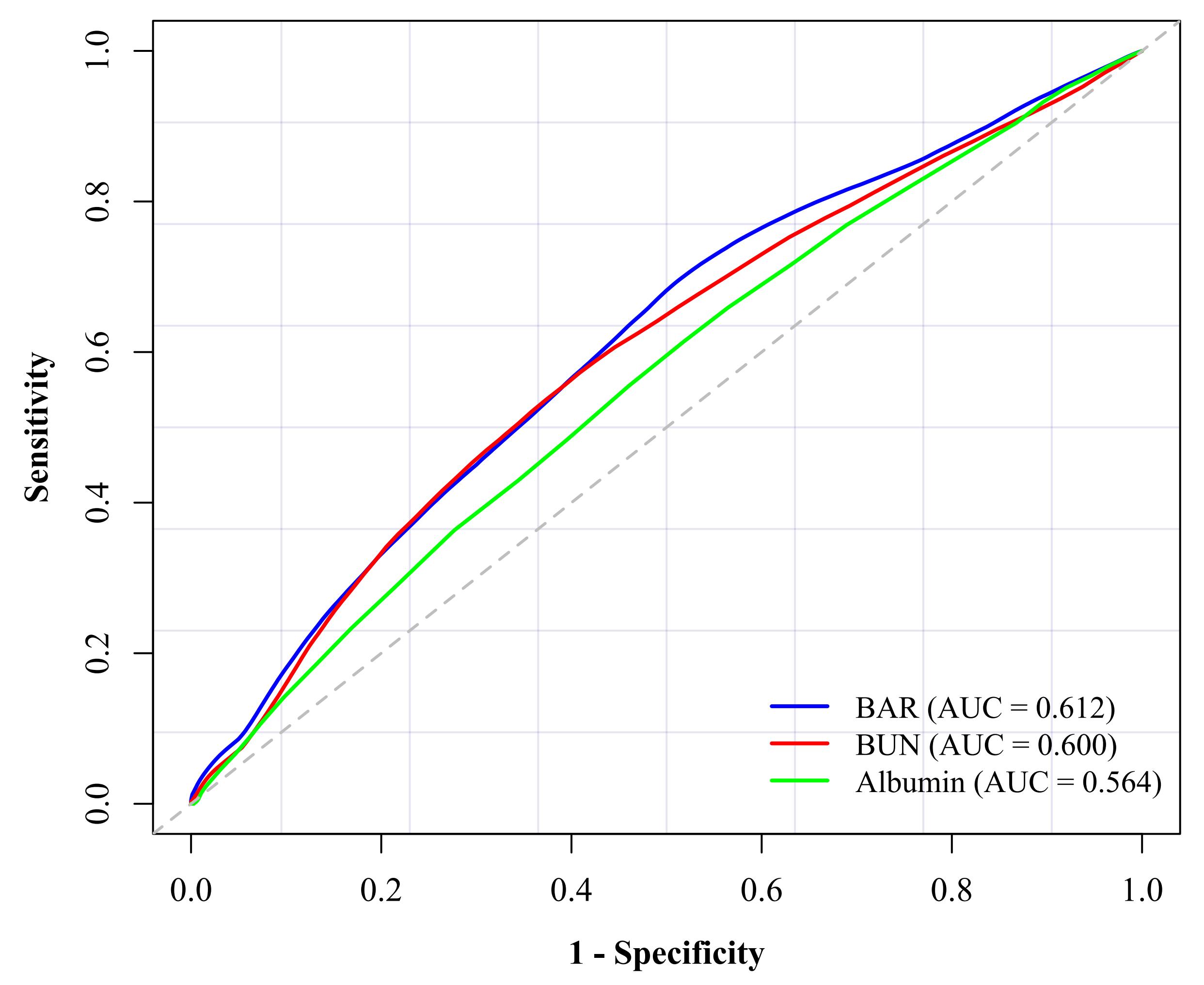 |
| --- |
| Figure S1 Time-dependent ROC curves of BUN, albumin and BAR |
